# Anterolateral thigh flap for contralateral adductor canal defects

**DOI:** 10.4103/0970-0358.73471

**Published:** 2010

**Authors:** Nikhil Panse, Parag Sahasrabudhe

**Affiliations:** Department of Plastic Surgery, B. J. Medical College and Sasoon Hospital, Pune, India

**Figure 1:**
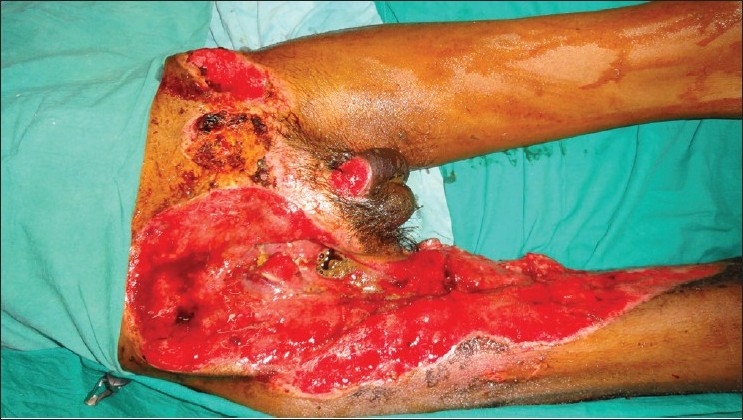
Exposed femoral vessels

**Figure 2:**
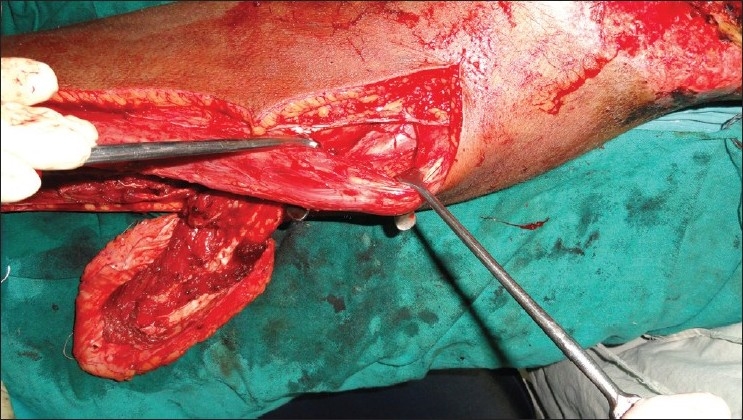
Selective tunneling of the rectus femoris

**Figure 3:**
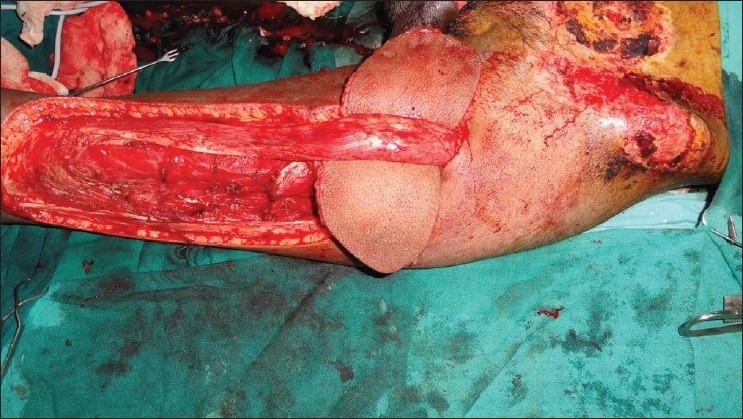
ALT being tunneled under the rectus femoris

Sir,

We read with great interest the article on AnteroLateralThigh flap for contralateral groin defects.[1] We congratulate the authors for elaborating the technique in great details, especially for its use for the contralateral groin defects. To what the authors have already stated, we would like to add the following:

1. We had a patient with exposed femoral vessels and pubic bone where we had the opportunity to use the contralateral ALT flap. The ALT reached up to the upper third of the contralateral adductor canal when vertically oriented, instead of the transverse orientation used to cover the groin defect. We used a similar technique of taking the pedicle over the suprapubic area. Late post op photographs were not available as the patient succumbed due to other comorbid conditions [Figures 1 2,3 4] The technique of medialising and lengthening the pedicle is already described in detail. However, the distal extent of the musculocutaneous flap, if extended to just above the knee, will further add to 10 more centimeters to the coverage area of the flap, and may reach up to the middle third of the adductor canal. The cutaneous territory of ALT may extend from the horizontal line at the level of the greater trocanter to the parallel line just above the level of patella, the centre of flap corresponding to perforating vascular bundle through aponeurosis at the junction of the upper and middle thirds of the thigh.[2]
2. The authors fear for vascular compromise of the rectus due to skeletonisation of the pedicle and tunneling the flap under the rectus. The rectus most of the times is a class II muscle with dominant branches from the descending branches of the lateral circumflex femoral artery. In addition to this, there are multiple vascular channels from the common trunk of the ascending and transverse branches of the lateral circumflex femoral artery and inferiorly from the branches of the femoral artery directly or via its branches to the vastus medialis.[3,4] We would also like to stress that during tunneling the flap underneath the rectus, it is necessary to make a tunnel sufficient enough only for the flap and keep the other attachments and perforators of the rectus intact so as to prevent vascular compromise [Figures 2 and 3].

**Figure 4:**
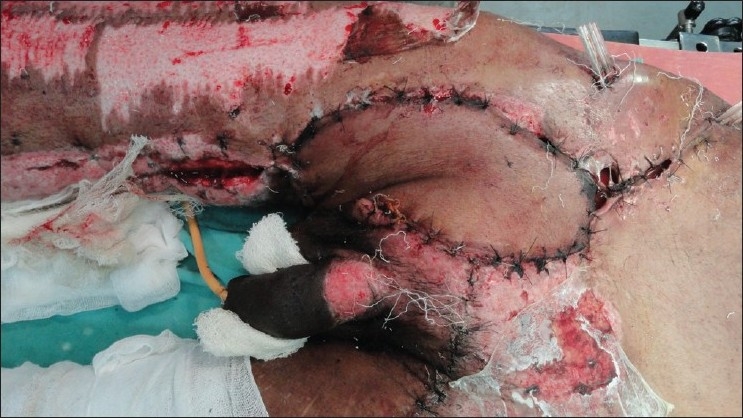
ALT reaching the contralateral adductor canal

To conclude, we would like to restate that ALT is a versatile flap and can be used to cover the defects of the contralateral adductor canal.
